# If you’re happy and you know it: neural correlates of self-evaluated psychological health and well-being

**DOI:** 10.1093/scan/nsad065

**Published:** 2023-11-01

**Authors:** Danielle Cosme, Arian Mobasser, Jennifer H Pfeifer

**Affiliations:** Department of Psychology, University of Oregon, Eugene, OR 97403, USA; Annenberg School for Communication, University of Pennsylvania, Philadelphia, PA 19104, USA; Department of Psychology, University of Oregon, Eugene, OR 97403, USA; Department of Psychology, University of Oregon, Eugene, OR 97403, USA

**Keywords:** positive neuroscience, wellness, social connectedness, self-reflection, vmPFC, pgACC

## Abstract

Psychological health and well-being have important implications for individual and societal thriving. Research underscores the subjective nature of well-being, but how do individuals intuit this subjective sense of well-being in the moment? This pre-registered study addresses this question by examining the neural correlates of self-evaluated psychological health and their dynamic relationship with trial-level evaluations. Participants (*N* = 105) completed a self-evaluation task and made judgments about three facets of psychological health and positive functioning—self-oriented well-being, social well-being and ill-being. Consistent with pre-registered hypotheses, self-evaluation elicited activity in the default mode network, and there was strong spatial overlap among constructs. Trial-level analyses assessed whether and how activity in a priori regions of interest—perigenual anterior cingulate cortex (pgACC), ventromedial prefrontal cortex (vmPFC) and ventral striatum—were related to subjective evaluations. These regions explained additional variance in whether participants endorsed or rejected items but were differentially related to evaluations. Stronger activity in pgACC was associated with a higher probability of endorsement across constructs, whereas stronger activity in vmPFC was associated with a higher probability of endorsing ill-being items, but a lower probability of endorsing self-oriented and social well-being items. These results add nuance to neurocognitive accounts of self-evaluation and extend our understanding of the neurobiological basis of subjective psychological health and well-being.

## Introduction

Much of our day-to-day lives are oriented toward being well. Importantly, research suggests that a more complete picture of psychological health includes not only the absence of disease and illness (World Health Organization, 1946) but also positive psychological functioning. Across many facets of life, the prospect of feeling good and functioning well is associated with broadly desirable outcomes—from higher wages (Staw *et al.*, 1994; Diener, 2000) and community engagement (Helliwell and Putnam, 2004; Helliwell, 2006) to lower divorce rates (Waite, 1995) and better health outcomes (Lyubomirsky *et al.*, 2005; Chida and Steptoe, 2008; Boyle *et al.*, 2010, 2012; Brand *et al.*, 2010; Haar and Roche, 2010). Despite continued debate about what constitutes the ‘good life’ or well-being, there is relative agreement that a person’s psychological health depends on their own subjective evaluation (Diener *et al.*, 2018b). But how do individuals intuit this subjective sense of well-being in the moment? Leveraging functional magnetic resonance imaging (fMRI) to address this question in a sample of late adolescents, this study examines the neural underpinnings of evaluating one’s own well-being and how these neural responses are dynamically related to perceptions of well-being in the moment.

### Multifaceted nature of well-being

Contemporary models of psychological health and well-being are derived from a variety of perspectives on what it means to be well, including feelings of happiness (Diener and Lucas, 1999), successful pursuits of personal goals (Seligman, 2011; Huppert and So, 2013; Butler and Kern, 2016) and satisfaction of fundamental psychological needs (Ryan and La Guardia, 2000; Ryan and Deci, 2001), among many others. These models tend to include or be nested within superordinate categories derived from ancient philosophical distinctions of hedonic well-being—consisting of pleasure or positive emotions (Kahneman *et al.*, 1999)—and eudaimonic well-being—which typically refers to the pursuit of meaningful goals and realization of one’s fullest potential (Waterman, 1993; Ryff, 1995). Notably, while the study of distinct forms of psychological health and well-being can help to improve the understanding of conceptual interrelations (Emmons, 1986; Csikszentmihalyi and Wong, 1991; McAdams and de St. Aubin, 1992), neural processes (Costa *et al.*, 2019) and intervention development (Lerner *et al.*, 2000; Seligman, 2018; Zeng and Kern, 2019), latent factors corresponding to the various models of psychological well-being are shown to be highly correlated with one another and exhibit poor discriminant validity (Disabato *et al.*, 2016; Longo *et al.*, 2016; Goodman *et al.*, 2017). Given that there are various theoretical models, each emphasizing different elements of well-being, and the practical constraints of fMRI task design, we adopted a theory-guided but data-driven approach to generate three factors related to psychological health and well-being that participants could evaluate while in the MRI scanner. Using factor analysis on a large pool of items from commonly used mental health and well-being measures, we identified three facets related to self-oriented well-being, social well-being and ill-being.

Although these facets are clearly related and are all indicators of psychological health and well-being, there is also evidence that they are meaningfully distinct (Headey *et al.*, 1993; Keyes and Ryff, 2003; Suldo and Shaffer, 2008; Gallagher *et al.*, 2009). For example, longitudinal evidence suggests that while older adults experience lower levels of mental illness (e.g. depression and anxiety) than younger adults, these groups do not differ in the level of well-being (Westerhof and Keyes, 2010). Among those with mental illness, variability in psychological functioning may be related to differences in well-being (Keyes, 2005). Additional evidence for the dissociability of well-being and ill-being comes from physiological research, indicating that the association between well-being and a given neuroendocrine or cardiovascular biomarker is generally not accompanied by significant, inverse associations between that same biomarker and ill-being (or vice versa; Ryff *et al.*, 2006). There is also evidence that social well-being is distinct from self-oriented well-being. Testing competing hierarchical models of well-being, Gallagher *et al.* (2009) identified that the best-fitting structural model of well-being included social well-being as a factor separate from hedonic and eudaimonic (self-oriented) well-being. In line with this, social connectedness is an important predictor of psychological health across the lifespan (Larson, 1993, 1996; Keyes, 1998) and perceived social support mitigates the effects of negative life events on well-being (Diener *et al.*, 2018a). In adults, marital satisfaction has been shown to buffer against day-to-day fluctuations in happiness more than time spent with others (Waldinger and Schulz, 2010). In adolescents, perceived social support is associated with greater life satisfaction (Suldo and Huebner, 2004), and longitudinally, adolescents’ satisfaction of relatedness needs is associated with later improvements in the quality of life (Gillison *et al.*, 2008). Taken together, these findings support the present investigation of self-oriented well-being, social well-being and ill-being as related but distinct facets of psychological health and well-being.

### Neuroscience of psychological health and self-evaluation

Given the strong connection between well-being and physical health, researchers have become increasingly interested in the neural correlates of well-being. This line of inquiry has focused primarily on how individual differences in self-reported well-being are related to measures of structural anatomy or resting state functional connectivity (Lewis *et al.*, 2014; Sato *et al.*, 2015; Kong *et al.*, 2015a,b, 2019; Luo *et al.*, 2016, 2017; Shi *et al.*, 2018; Maeda *et al.*, 2021) or brain activity while processing affective stimuli (van Reekum *et al.*, 2007; Waldinger *et al.*, 2011; Heller *et al.*, 2013; Morelli *et al.*, 2018; Puccetti *et al.*, 2021). However, to understand how individuals intuit and evaluate their own, subjective sense of well-being in the moment requires the examination of dynamic relationships between well-being evaluations and brain activity within persons. Trial-level analyses in which brain signals within regions of interest (ROIs) are extracted and used to predict task-related behavior are becoming increasingly common for studying such dynamic brain–behavior relationships within persons (Cosme *et al.*, 2019; Shenhav and Karmarkar, 2019; Barendse *et al.*, 2020; Wilson *et al.*, 2021).

Despite a robust body of literature on the neural correlates of self-evaluation in response to trait words, we are unaware of any studies assessing neural responses during self-evaluation of subjective psychological health. This is surprising given that the self-reflective process of evaluating one’s own well-being may be directly implicated in psychological health. During self-evaluation, adults and adolescents reliably engage cortical midline structures (CMSs), including perigenual anterior cingulate cortex (pgACC), anterior rostral/ventral medial prefrontal cortex (mPFC), and medial posterior parietal cortex (mPPC), and ventral striatum (VS) as well (Northoff and Hayes, 2011; Denny *et al.*, 2012; Pfeifer and Berkman, 2018). Much of the work in this field has used relatively neutral traits, although some have used stimuli with explicitly positively or negatively valenced traits (Lemogne *et al.*, 2009; Frewen *et al.*, 2013; Chavez *et al.*, 2017; Barendse *et al.*, 2020; Elder *et al.*, 2023) or positive and negative feedback (van Schie *et al.*, 2018). Furthermore, most studies used personality traits that were sampled broadly without considering the self-concept domain. Those that have assessed specific domains found differences in the degree of CMS involvement when comparing across domains (Moran *et al.*, 2006; Jankowski *et al.*, 2014; van der Cruijsen *et al.*, 2017, 2018), but the domains investigated vary across studies, making it difficult to identify consistencies in these results.

Nevertheless, there are general patterns described in the literature of what can be considered representational gradients—particularly within the mPFC—that may help guide predictions about how an individual’s sense of subjective psychological health is constructed in the brain. Trait evaluations that are oriented more toward self and close others are typically represented more ventrally than those referencing distant others (Mitchell *et al.*, 2005). The mPFC is also implicated in future-oriented and abstract thought, including prospection (Spreng and Grady, 2010). Subjective value is likewise represented in the vmPFC (Clithero and Rangel, 2014), which may also reflect personal relevance (Kim and Johnson, 2015; Doré *et al.*, 2019). Given that psychological health consists of multiple facets that vary in degree of sociality, abstractness, future orientation and positive or negative affect, it is plausible that there is meaningful variation across constructs in the neural representation of an individual’s sense of subjective well-being.

### The present study

Building on these findings, the goal of the current study was to investigate the neural basis for how individuals construct and access their own subjective sense of well-being in the moment. While in the MRI scanner, participants completed a self-evaluation task and made judgments about three facets of psychological health and positive functioning: self-oriented well-being (e.g. positive affect, life satisfaction, competence and meaning), social well-being (e.g. relatedness, belonging and social support) and ill-being (e.g. anxiety, depression and anger). We conducted pre-registered (i) whole-brain conjunction and contrast analyses to identify the similar and distinct neural correlates of evaluating these facets of psychological health and (ii) multilevel logistic regression analyses to test whether activity in a priori ROIs implicated in general self-evaluation and affective processing—pgACC, vmPFC and VS—is dynamically associated with trial-level subjective evaluations of well-being.

Based on previous findings on the neural basis of self-referential processing, we expected to see robust activity in CMS and sub-CMS, including pgACC, vmPFC and VS, during self-evaluation relative to a control condition. However, we also explored how these different constructs associated with psychological health (self-oriented well-being, social well-being and ill-being) may be represented in both similar and distinct ways. In trial-level analyses, we expected that neural predictors would explain additional variance beyond behavior. We hypothesized that the vmPFC would be related to endorsements across all constructs (self-oriented well-being, social well-being and ill-being) due to its role in valuation and decision-making. Similarly, because the pgACC is implicated in self-referential processing across a variety of domains, we expected that the pgACC would also be related to endorsements regardless of constructs. Although we did not have strong hypotheses regarding the distinctive effects of the VS across constructs, given its central role in reward processing (Haber, 2011), one possibility is that the VS would track valence such that greater activity in the VS is related to endorsements of self-oriented and social well-being and rejections of ill-being.

## Methods

### Open practices statement

The analysis code and behavioral and trial-level ROI data are available online (https://github.com/dsnlab/happy_scripts). Unthresholded statistical maps for this effect and all others reported in this paper can be found on NeuroVault (Gorgolewski *et al.*, 2015; https://neurovault.org/collections/NWIKSNNW). Our hypotheses and analysis plan were pre-registered (https://osf.io/x2zra). However, we deviated from this analysis plan in several places. First, we originally planned to model item valence and well-being constructs as simultaneous predictors, but this made the interpretation of model parameter estimates challenging because only the social well-being construct included both positive and negative items, whereas self-oriented well-being included only positive items and ill-being included only negative items. In the main paper models, we removed valence and reverse-coded the four negative items in the social well-being construct so that rejections of negative items were coded as positive endorsements of social well-being. We report models including valence only in the Supplementary material. Second, in response to reviewer feedback, we accounted for differences in processing time between constructs by including log-transformed reaction time in the trial-level analyses. Models without reaction time are included in the Supplementary material. Third, we originally planned to conduct trial-level representational similarity analyses, but the task was not appropriately optimized for this and resulted in unstable estimates. For completeness, we report the results in the Supplementary material. Finally, we pre-registered individual difference analyses but given the within-person focus of this paper, we report these in the Supplementary material.

### Participants

Participants were 113 incoming freshmen (0 non-binary, 41 men and 72 women) at the University of Oregon enrolled in a longitudinal study on health and well-being during the transition to college. Detailed information about recruitment and inclusion criteria is provided in the Supplementary material. We excluded participants if they did not comply with task instructions (*n* = 4) or had usable responses (*n* = 1), or if they had missing data due to a technical failure (*n* = 1) or exhibited excessive head motion (*n* = 1). Participant compliance was assessed by asking participants to explain the instructions and how they did the task after the MRI scan was completed. Two participants were excluded from whole-brain analyses because they did not complete both task runs. One participant was excluded from trial-level analyses conducted in native space because we were unable to collect a T1-weighted anatomical image to create individualized ROIs. Because the trial-level analysis could include the data from participants with a single task run, two participants excluded from the whole-brain analyses were included in this analysis in order to reduce bias. This yielded a total of 104 participants for whole-brain analyses and 105 participants for the trial-level analysis. This study was approved by the University of Oregon Institutional Review Board; all participants gave written informed consent and were compensated for their participation. Demographic information is reported in the Supplementary material.

### Sample demographics

After exclusions, the final sample included: 0 non-binary participants, 37 men and 68 women. All participants were 17–19 years old (*M* = 18.02, s.d. = 0.24). Of participants, 25% (*N* = 26) identified as first-generation college students. The racial and ethnic composition and subjective social status of the sample are reported in Table 1. Participants self-reported their race and ethnicity using the following categories based on the National Institutes of Health policy: American Indian or Alaska Native, Asian, Black or African American, Hispanic or Latino/Latina/Latinx, Native Hawaiian or other Pacific Islander and White. With respect to race and ethnicity, our sample was largely representative of the 2018 University of Oregon freshman class as a whole, which reported the following racial and ethnic identities: 7% Asian American, 3% Black or African American, 15% Hispanic or Latino/Latina/Latinx, 9% Multiethnic, 0.5% Native American, 0.7% Native Hawaiian or other Pacific Islander and 64% White.

**Table 1.** Racial and ethnic composition and subjective social status of the sample

	Hispanic or Latina/Latino/Latinx			
	Yes		No	
	*N*	%	*N*	%
Race				
Asian			10	9.6
Black or African American			3	2.9
More than one race	6	5.8	9	8.7
Native Hawaiian or other Pacific Islander			1	1.0
Prefer not to state	4	3.8		
White	10	9.6	62	59.6
Subjective social status				
Not enough money to get by	1	1.0		
Just enough money to get by	31	29.8		
We only have to worry about money for fun or extras	56	53.8		
We never have to worry about money	17	16.3		

### Self-evaluation task

**Fig. 1.**
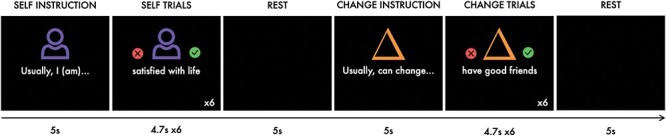
Self-evaluation task design consisting of six alternating self and control (change) blocks per run. Each block began with a 5 s instruction, followed by six words or phrases, each of which was presented for 4.7 s. Each trial was followed by a jittered black screen for ∼0.3 s and a black screen was presented for 5 s between blocks.

While being scanned, participants completed a self-evaluation task (adapted from Moore, 2015; available online at https://github.com/dsnlab/svc-wellbeing), in which they alternated between making self-evaluations (‘self’ condition) and judging the malleability (‘change’ condition) of words or short phrases related to three constructs associated with psychological health and positive functioning: self-oriented well-being, social well-being and ill-being. These constructs and the items within each construct were selected from a large pool of items (*N *= 170) from psychological health questionnaires using factor analysis (Supplementary material). Example items in each of the constructs include happy, satisfied with life and joyful (self-oriented well-being); loved, feel supported and cared for (social well-being); and anxious, angry and feel worthless (ill-being). The full list of items for each construct can be found in the Supplementary material. In the self condition, participants reported whether each of the stimuli described themselves; in the control (change) condition, participants reported whether each item was something that could change for people in general. The experimental task (Figure 1) was a 2 × 3 repeated measures factorial ANOVA design, with the following factors and levels: Instruction (self or change) and Construct (self-oriented well-being, social well-being or ill-being).

This task was administered across two runs and each run contained 36 trials. Stimuli were presented and responses were recorded in MATLAB 2017b (MathWorks, Inc., Natick, MA, USA), using the Psychtoolbox 3 (Brainard, 1997; Kleiner *et al.*, 2007). The task was a mixed design (i.e. a cross between an event-related and block design) in which items from each construct were nested within alternating self and change blocks. Each word or phrase was presented once per run in either the self or change condition. To facilitate generalizability beyond the specific stimuli used in the task (Westfall *et al.*, 2017), each participant saw 12 items from each construct randomly selected from a larger set of 18 items per construct. Participants responded ‘yes’ or ‘no’ to each item using a button box. Response keys (index or middle finger) were counterbalanced across participants. Within each run, the trial order was optimized to maximize event-related contrast estimation using a genetic algorithm (Wager and Nichols, 2003).

### Neuroimaging data acquisition and preprocessing

Neuroimaging data were acquired on a 3T Siemens Skyra scanner at the University of Oregon Lewis Center for Neuroimaging. Scan sequence information is listed in the Supplementary material. Neuroimaging data were preprocessed using fMRIPrep 1.1.4 (Esteban *et al.*, 2019). Preprocessing details appear in the Supplementary material, but briefly, anatomical images were segmented and normalized to Montreal Neurological Institute (MNI) space using FreeSurfer (Fischl, 2012); functional images were susceptibility distortion corrected, realigned and coregistered to the normalized anatomical images. For whole-brain analyses in MNI space, preprocessed functional data were smoothed using a 6 mm full-width at half maximum (FWHM) smoothing kernel; for ROI analyses in native space, images were smoothed with a 2 mm FWHM smoothing kernel.

### Whole-brain main effects

In first-level statistical analyses, event-related condition effects were estimated using a fixed-effects general linear model and convolving the canonical hemodynamic response function with stimulus events using SPM12 (Wellcome Department of Cognitive Neurology; http://www.fil.ion.ucl.ac.uk/spm). Separate regressors were entered for conditions of interest (self self-oriented well-being, self social well-being, self ill-being, change self-oriented well-being, change social well-being change ill-being). Event duration was modeled as stimulus onset to response. Across trials, the mean reaction time was 1.6 s (Med = 1.5 s, s.d. = 0.6 s), meaning that there was on average 3.4 s between events. Trials in which no response was made were modeled as a separate condition of no interest and each missed event was modeled as the full duration of stimulus presentation (4.7 s). An additional regressor was included for the instruction period at the beginning of each block. Realignment parameters were transformed into five motion regressors, including absolute displacement from the origin in Euclidean distance and the displacement derivative for translation and rotation separately, and a single ‘trash’ regressor for images with motion artifacts (i.e. striping) identified using automated motion assessment via the confounds file generated by fMRIPrep (Cosme *et al.*, 2018) and visual inspection. These regressors were included as covariates of no interest. One participant was excluded from the group-level analysis for having >15% unusable volumes, which was more than 5 s.d. from the mean (*M* = 1.47%, s.d. = 2.70%). All data were high-pass filtered at 100 s and temporal autocorrelation was modeled using the FAST method (Corbin *et al.*, 2018). Linear contrasts for each condition of interest *vs* rest were estimated across runs for each participant and used as inputs in second-level analyses.

The second-level main effect of self *vs* change was modeled as a 2 × 3 within subject repeated measures ANOVA using a flexible factorial model in SPM12. To correct for multiple comparisons, cluster-extent thresholding was implemented using AFNI version 18.2.04 (Cox, 1996). In accordance with recent guidelines (Cox *et al.*, 2017), the spatial autocorrelation function was first estimated for each subject and task run separately using AFNI’s 3dFWHMx and then averaged across subjects. To determine probability estimates of false-positive clusters given a random field of noise, Monte Carlo simulations were conducted with AFNI’s 3dClustSim using the average autocorrelation across subjects. A voxel-wise threshold of *P* < 0.0001 and a cluster extent of *k* > 42 were estimated (voxel dimensions = 2 mm^3^) to achieve a whole-brain family-wise error rate of α = 0.01.

### Conjunction and contrast analyses

To assess the degree of spatial overlap between the three constructs of psychological health, we compared activation maps for self-oriented well-being self > self-oriented well-being change, social well-being self > social well-being change and ill-being self > ill-being change, separately. Because it is not possible to retrieve these contrasts from the flexible factorial model used in the self > change analysis, we generated these contrast maps using one-sample *t*-tests in SPM12. We then thresholded each contrast map using the same approach described earlier and qualitatively compared spatial representations by overlaying these images on one another. For completeness, we also conducted exploratory contrast analyses to determine the unique self > change effects of each construct with respect to the other two (e.g. self-oriented well-being self > ill-being and social well-being self > self-oriented well-being change > ill-being and social well-being change). These contrasts were extracted from the flexible factorial model.

### ROI definition

**Fig. 2.**
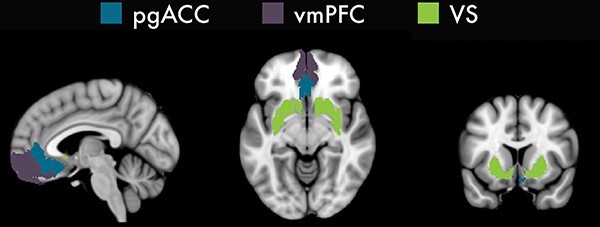
A priori ROIs used in the similarity, trial-level and individual difference analyses. ROIs were created using the HCP MMP 1.0 cortical parcellation atlas (Glasser *et al.*, 2016) and FreeSurfer subcortical segmentation atlas (Fishl *et al.*, 2002).

We defined three a priori bilateral ROIs for the pgACC, vmPFC and VS (Figure 2) using the HCP MMP 1.0 cortical parcellation atlas (Glasser *et al.*, 2016). Further details describing the definition process are provided in the Supplementary material.

### Trial-level analysis

To investigate the extent to which activity in brain regions associated with self-evaluation and affective processing (i.e. pgACC, vmPFC and VS) is related to item-level endorsements, we used multilevel logistic regression with neural data from each trial. First-level statistical analyses were conducted in native (i.e. non-normalized) space. Each trial was entered into the model as a separate regressor (rather than grouped by condition). Trial duration was specified as described in the univariate contrast analysis section, and the same motion parameter modeling procedure was adopted. The resulting statistical maps for each trial were concatenated to create a beta series (Rissman *et al.*, 2004). To calculate the mean blood-oxygen-level-dependent (BOLD) signal across the voxels in each ROI, we use the 3dmaskave function in AFNI 18.2.04 (Cox, 1996). For each participant, we extracted the mean parameter estimate of the BOLD signal within each ROI for each trial in the beta series. Parameter estimates were standardized within participant and ROI to account for differences in variability between individuals and ROIs. Outliers that were 3 s.d. from the median (*N* = 324 or 0.74% of trials) were excluded and only trials within the self condition were modeled.

We conducted multilevel logistic regression in R using the glmer function in the lme4 package (Bates *et al.*, 2015). In a series of nested models, trial-level item endorsements (yes or no) were regressed on the fixed effects of construct (well-being, ill-being or social), ROI (pgACC, vmPFC and VS), the interactions between construct and ROI, grand-mean centered log-transformed reaction time and the interactions between construct and reaction time. Intercepts were allowed to vary randomly across participants. We estimated the following nested models and compared model fit using chi-squared difference tests. All ROIs were included in the same model in order to assess their unique relationships with endorsements. We report parameter estimates and statistics from the best-fitting model. We converted log-odds parameter estimates to marginal probabilities using the ggeffects package (Lüdecke, 2018) and estimated multilevel model effect sizes by calculating *R*^2^ according to the guidelines in Lorah (2018) using the performance package (Lüdecke *et al.*, 2021). To estimate descriptive correlations between ROIs and account for the nested structure of trials within participants, we calculated repeated measures correlations using the rmcorr package (Bakdash and Marusich, 2017). We conducted separate sensitivity analyses reported in the Supplementary material (i) modeling valence instead of well-being construct, (ii) including the change condition and (iii) including item as a random effect.

## Results

### Whole-brain main effects

**Fig. 3.**
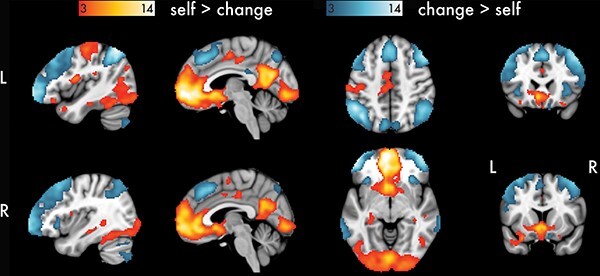
Univariate main effects for instruction. Results are thresholded at *P* < 0.0001 and *k* = 42. Cluster extent (*k*) is measured in 2 mm^3^ voxels.

To investigate brain regions that were associated with self-evaluation of psychological health and well-being, we contrasted the BOLD signal between the self and change conditions (Figure 3). Consistent with prior studies on self-referential processing and self-evaluation, for self > change, we observed robust BOLD signal increases in regions within the default mode network. This included CMSs, such as bilateral pgACC, subgenual ACC (sgACC), vmPFC, medial orbitofrontal cortex (mOFC) and posterior parietal cortex (PCC); lateral cortical regions, including inferior parietal lobule (IPL) and middle temporal gyrus (MTG); and subcortical regions, such as bilateral VS and hippocampus. For change > self, we observed increased BOLD signal in regions within the frontoparietal control network, such as bilateral ventrolateral and dorsolateral prefrontal cortex, and PPC; and the salience network, including bilateral dorsal anterior cingulate cortex and anterior insula. All clusters and peak coordinates are listed in (Table 2). Contrast tables were generated using BSPMVIEW (Spunt, 2016).

**Table 2.** Regions, MNI coordinates, cluster extent and peak *t* values for the main effects of instruction

Contrast	Region	MNI coordinates (*x*, *y*, *z*)			Extent (*k*)	Peak *t*
Self > change	L mid orbital gyrus	−4	60	−6	8186	15.87
	R rectal gyrus	8	40	−10	8186	12.94
	L olfactory cortex	−4	16	−6	8186	11.23
	L precuneus	−6	−64	20	11 730	12.12
	L calcarine gyrus	−2	−86	−6	11 730	10.11
	R lingual gyrus	16	−100	−2	11 730	6.91
	L lingual gyrus	−16	−90	−10	11 730	3.98
	L rolandic operculum	−44	−4	16	2656	6.84
	L precentral gyrus	−40	−18	58	2656	6.76
	L postcentral gyrus	−60	−20	26	2656	6.32
	L hippocampus	−24	−22	−14	520	6.33
	L inferior temporal gyrus	−44	−24	−20	520	5.13
	L parahippocampal gyrus	−24	−6	−28	520	4.01
	R inferior frontal gyrus (pars orbitalis)	26	16	−20	76	6.29
	R superior temporal gyrus	66	−34	26	754	6.27
	L MCC	−6	−6	50	527	6.22
	R hippocampus	26	−18	−14	303	6.17
	R parahippocampal gyrus	38	−44	0	303	4.70
	L middle temporal gyrus	−64	−10	−12	272	6.15
	R middle temporal gyrus	60	−6	−14	127	5.75
	R precentral gyrus	56	4	38	71	5.65
	L IFG (pars orbitalis)	−40	30	−16	124	5.59
	L superior frontal gyrus	−18	38	36	128	5.08
	R insula lobe	44	0	16	62	4.96
	R posterior medial frontal	6	−4	60	49	4.79
	L middle occipital gyrus	−42	−78	32	64	4.75
	R superior temporal gyrus	48	−28	−2	89	4.44
Change > self	R middle temporal gyrus	72	−28	−14	848	3.95
	L inferior parietal lobule	−52	−52	54	2215	14.89
	L superior parietal lobule	−26	−72	62	2215	4.48
	L angular gyrus	−60	−64	30	2215	4.35
	L middle orbital gyrus	−46	54	−4	4652	13.78
	L middle frontal gyrus	−46	28	40	4652	10.56
	L middle frontal gyrus	−30	18	62	4652	9.93
	R inferior parietal lobule	48	−52	54	1821	13.01
	R middle orbital gyrus	44	56	−2	5918	12.59
	R middle frontal gyrus	46	24	46	5918	12.14
	R medial frontal gyrus	22	50	−16	5918	6.82
	L superior medial gyrus	0	34	44	1483	10.58
	L posterior medial frontal	−4	16	52	1483	8.22
	L inferior temporal gyrus	−62	−42	−20	1040	9.62
	R IFG (pars orbitalis)	32	28	−2	297	9.06
	L precuneus	−6	−72	48	685	8.14
	L IFG (pars orbitalis)	−32	26	−4	153	7.96
	R cerebellum (Crus 2)	10	−82	−26	87	7.00
	R inferior temporal gyrus	68	−30	−20	848	6.72
	R middle temporal gyrus	66	−44	−2	848	4.56
	L cerebellum (Crus 2)	−12	−82	−26	110	6.50
	L superior frontal gyrus	−18	50	−20	179	6.31
	R cerebellum (Crus 1)	30	−64	−32	204	6.19
	R cerebellum (Crus 2)	42	−76	−46	366	6.15
	R superior orbital gyrus	12	14	−18	90	5.98
	L cerebellum (Crus 2)	−40	−76	−48	240	5.83
	L PCC	−6	−30	26	68	4.92

*Notes*: Cluster family-wise error correction for α = 0.01 and *P* < 0.0001 is *k* = 42. Cluster extent (*k*) is measured in 2 × 2 × 2 mm voxels.

### Conjunction and contrast analyses

**Fig. 4.**
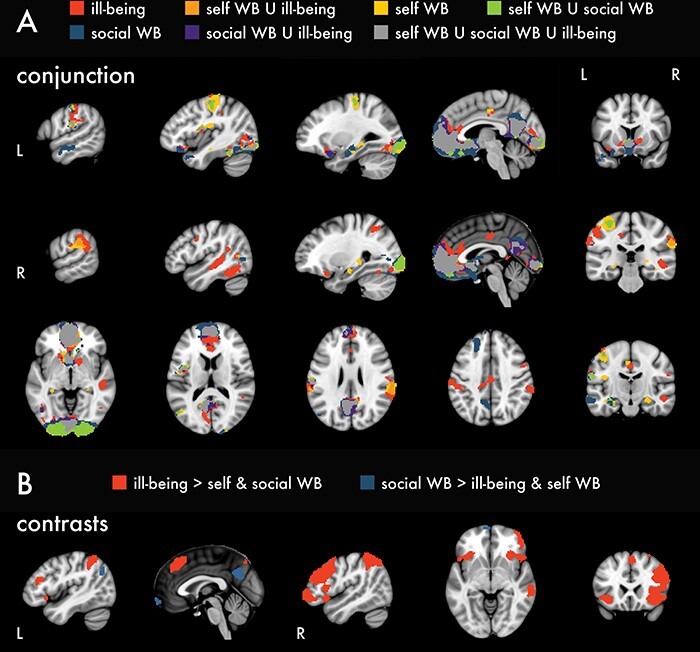
Conjunction and contrast analyses. (A) Conjunction analysis for self > change within each construct separately. (B) Contrasts for the interaction between self > change and each construct > the average of the other two. No clusters survived thresholding for self-oriented well-being > ill-being and social well-being. Contrasts were thresholded at *P* < 0.0001, *k* = 42 (voxels are 2 × 2 × 2 mm). WB = well-being.

We assessed spatial similarity among psychological health constructs for the self > change contrast by conducting a statistical conjunction analysis, interpreted qualitatively via visualization in (Figure 4A). On the whole, we observed strong spatial overlap among constructs within core regions involved in self-evaluation, such as pgACC, sgACC, vmPFC, mOFC, PCC and VS. Ill-being items were associated with the expansion of spatial extent into more dorsal aspects of the mPFC, as well as the presence of additional clusters of activation in regions such as left inferior frontal gyrus, bilateral midcingulate cortex and right MTG, fusiform gyrus and temporal parietal junction. However, exploratory contrasts revealed that only the clusters in the left inferior frontal gyrus and right MTG were actually unique to ill-being (Figure 4B). For social well-being items, activation extended into more anterior and ventral regions in the mPFC and more dorsal regions in the PCC and precuneus and was associated with additional clusters of activation in the left MTG and anterior temporal cortex. The clusters in the anterior mPFC, left MTG and precuneus were unique to social well-being items. Clusters of activation associated with self-oriented well-being items showed expanded spatial extent in the left posterior insula and postcentral gyrus, and right hippocampus, but these were not unique to self-oriented well-being.

### Trial-level analysis

We used multilevel logistic regression to investigate the degree to which psychological health construct (self-oriented well-being, social well-being ill-being) and the mean BOLD signal in a priori ROIs—pgACC, vmPFC and VS—were associated with whether participants answered yes or no to each item in the self-evaluation task. ROI descriptive statistics are reported in (Table 3). We tested a series of nested models to determine whether adding neural predictors explained additional variance beyond a base model, which included construct (self-oriented well-being, social well-being and ill-being), log-transformed reaction time and their interaction. As expected, the best-fitting model was the ROI interaction model, which included the ROIs and their interactions with constructs (Table 4). This model fit the observed data well (*R*^2^ = 0.53).

**Table 3.** Means, s.d., and repeated measures correlations among ROIs

ROI	*M*	s.d.	1	2	3
1. pgACC	0.04	1.03			
2. vmPFC	0.15	1.02	0.84 (0.83, 0.85)		
3. VS	0.21	1.03	0.41 (0.39, 0.45)	0.45 (0.43, 0.48)	

*Notes*: *N* = 3694 trials in the self condition. All correlations are statistically significant, *P* < 0.001. 95% CIs are in parentheses. Correlations adjust for trials nested within participants using multilevel modeling (df = 3588).

**Table 4.** Model comparison of trial-level models

	AIC	BIC	χ^2^	df	*P*
Base model	3113.82	3157.32			
ROI model	3100.49	3162.63	19.34	3	<0.001
**ROI interaction model**	**3082.28**	**3181.71**	**30.21**	**6**	**<0.001**

*Notes*: The best fitting model is bolded.

**Fig. 5.**
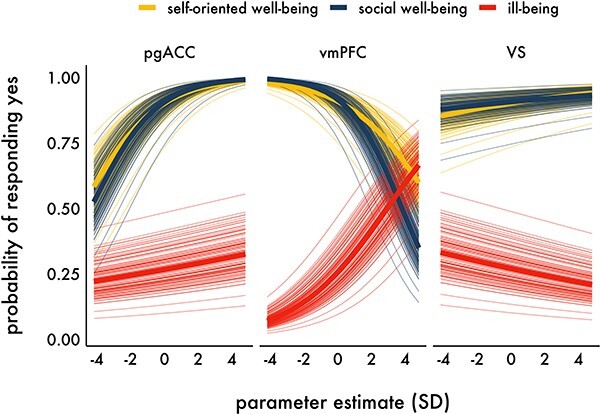
Predicted probabilities from the ROI interaction model as a function of construct. Thinner lines indicate the predicted probabilities for each participant as a function of construct and ROI.

As expected (Fig. 5), there was a high probability of endorsing positively valenced self-oriented well-being [marginal *P* = 0.90, 95% confidence interval (95% CI) 0.88–0.92] and social well-being items (marginal *P* = 0.91, 95% CI 0.88–0.93), and a low probability of endorsing negatively valenced ill-being items (marginal *P* = 0.27, 95% CI 0.24–0.30). Greater than the average pgACC activity (i.e. a 1 s.d. increase) was associated with a higher probability of endorsing self-oriented well-being (marginal *P* = 0.93, 95% CI 0.90–0.96), social well-being items (marginal *P* = 0.94, 95% CI 0.91–0.96) and ill-being items (marginal *P* = 0.28, 95% CI 0.22–0.34), although this relationship was weaker for ill-being items. In contrast, greater than the average vmPFC activity was associated with a higher probability of endorsing ill-being items (marginal *P* = 0.33, 95% CI 0.27–0.39), but with a lower probability of endorsing self-oriented well-being (marginal *P* = 0.87, 95% CI 0.82–0.91) and social well-being items (marginal *P* = 0.85, 95% CI 0.80–0.89). Greater than the average VS activity was associated with directional increases in the probability of endorsing self-oriented well-being (marginal *P* = 0.91, 95% CI 0.88–0.93) and social well-being items (marginal *P* = 0.91, 95% CI 0.88–0.93), and decreases for ill-being items (marginal *P* = 0.26, 95% CI 0.22–0.30), but these relationships were not statistically significant. Log-odds and statistics for all parameters in the model are included in Table 5.

**Table 5.** Parameter estimates and statistics for the model including log-transformed reaction time

Parameter	*b*	*z*	*P*
Intercept (self-oriented well-being)	1.89 (1.68, 2.11)	16.93	<0.001
RT	−3.16 (−3.74, −2.57)	10.52	<0.001
Construct (ill-being)	−2.95 (−3.20, −2.70)	23.58	<0.001
Construct (social well-being)	0.09 (−0.20, 0.37)	0.59	0.552
pgACC	0.45 (0.08, 0.82)	2.37	0.018
vmPFC	−0.39 (−0.76, −0.02)	2.09	0.037
VS	0.11 (−0.11, 0.33)	0.98	0.328
RT × construct (ill-being)	3.16 (2.46, 3.86)	8.89	<0.001
RT × construct (social well-being)	−0.03 (−0.82, 0.75)	0.08	0.935
Construct (ill-being) × pgACC	−0.39 (−0.83, 0.05)	1.73	0.083
Construct (social well-being) × pgACC	0.07 (−0.44, 0.57)	0.26	0.792
Construct (ill-being) × vmPFC	0.76 (0.32, 1.20)	3.35	<0.001
Construct (social well-being) × vmPFC	−0.23 (−0.75, 0.29)	0.88	0.381
Construct (ill-being) × VS	−0.18 (−0.45, 0.09)	1.32	0.187
Construct (social well-being) × VS	−0.04 (−0.34, 0.27)	0.24	0.811

*Notes*: Parameter estimates (*b*) are log-odds. Reaction Times (RTs) were grand-mean centered and units are in seconds. Neural ROI parameter estimates were standardized within participants and ROIs to preserve individual differences and are therefore not *z*-scores.

## Discussion

Psychological health and well-being have important implications for individual and societal thriving. Research underscores the subjective nature of well-being, but how do individuals intuit this subjective sense of well-being in the moment? The present study addressed this question by examining the neural correlates of self-evaluated psychological health and their dynamic relationship with trial-level evaluations of well-being. We focused on three constructs related to psychological health: self-oriented well-being, social well-being and ill-being. We observed robust activity in CMS and sub-CMS during self-evaluation relative to the control condition. The spatial profiles of the constructs were largely overlapping, although evaluating ill-being was associated with relatively greater activity in the salience and frontoparietal networks. Trial-level analyses assessed whether and how activity in brain regions associated with affect and self-referential processing were related to subjective evaluations. Trial-specific neural activity in the pgACC, vmPFC and VS explained additional variance in whether participants endorsed or rejected items, and activity in the pgACC and vmPFC was differentially related to endorsements on a trial-by-trial basis. Specifically, stronger activity in the pgACC was associated with a higher probability of endorsement across constructs, whereas stronger activity in the vmPFC was associated with a higher probability of endorsing ill-being items, but a lower probability of endorsing self-oriented and social well-being items. Together, these results suggest that subjective sense of well-being is intuited using the same neural architecture underlying other forms of self-referential processing, but highlight the differential roles of the pgACC and vmPFC.

### Neural correlates of self-evaluating psychological health and well-being

Given the robust body of research on the neural correlates of self-referential processing, we had strong predictions that compared to the control condition, self-evaluation of psychological health would elicit increased neural activity in cortical and sub-CMS. In line with this prediction, participants showed relatively greater BOLD signal in bilateral pgACC, sgACC, vmPFC, mOFC, PCC and VS. In this well-powered sample, we also observed increased activity in bilateral hippocampus, and lateral cortical areas, including MTG and IPL. In general, these results are consistent with prior self-evaluation studies in adolescents using a mix of positively and negatively valenced words or phrases as stimuli (Pfeifer *et al.*, 2013; Jankowski *et al.*, 2014; van der Cruijsen *et al.*, 2018; Elder *et al.*, 2023). Although these patterns were relatively consistent across psychological health constructs, the spatial conjunction analysis showed that ill-being items were associated with more dorsal extension of activation in the mPFC, whereas social well-being items elicited more ventral extension of responses in the mPFC. In addition, exploratory construct contrasts revealed relatively greater activation in the salience and frontoparietal networks for ill-being items. Although previous self-evaluation studies examining valence have tended to report greater activation for positive relative to negative traits in the vmPFC (Crone *et al.*, 2022) but less consistent spatial specificity for negative relative to positive traits, the present results are consistent with findings from studies examining affective responses to positive and negative social feedback about the self (Frewen *et al.*, 2013; van Schie *et al.*, 2018). Thus, greater activation in these regions for ill-being items may reflect differences in valence or indicate greater conflict or cognitive control demands (Botvinick *et al.*, 2004). Despite these minor differences, the overall similarity with prior research on self-evaluation is notable given that these studies have focused almost exclusively on personal traits, rather than more primary, innate characteristics related to the self, as studied here. Seeking to be well is a fundamental motive and as such may be inextricably linked to the self (Northoff and Hayes, 2011; Chavez *et al.*, 2017). To advance our understanding of how the self is represented in the brain and develops over time, we hope future studies will employ a wider variety of stimuli, task designs (Qin *et al.*, 2020) and modeling approaches (Elder *et al.*, 2023).

### Within-person self-evaluation dynamics and the dissociable roles of the pgACC and vmPFC

Extending these whole-brain analyses, we novel modeling approach to examine how specific a priori brain regions associated with self-evaluation and affective processing (pgACC, vmPFC and VS) were dynamically related to subjective evaluations of well-being on a trial-by-trial basis. Despite not having strong predictions about the role of the VS in self-evaluation, we reasoned that due to its role in affective processing, the VS might track item valence and found directional (but not statistically significant) evidence for this. We expected that both pgACC and vmPFC would track evaluations on a trial-by-trial basis. Greater than the average pgACC activity was associated with an increased probability of responding yes across all psychological health constructs. In contrast, while the vmPFC also tracked responses, greater than the average vmPFC was associated with a higher probability of endorsing ill-being items, but a lower probability of endorsing self-oriented and social well-being items (i.e. a higher probability of rejecting them). These results are consistent with previous findings that activity in the vmPFC predicts choices on a trial-by-trial basis (Engelmann *et al.*, 2015; Cosme *et al.*, 2019; Shenhav and Karmarkar, 2019; Barendse *et al.*, 2020; Wilson *et al.*, 2021) and that stronger activity in the pgACC and vmPFC is associated with a higher probability of endorsing negatively valenced traits as self-descriptive (Frewen *et al.*, 2013; Barendse *et al.*, 2020). However, the pattern of results in the vmPFC—that is, a higher probability of endorsing negative ill-being items and rejecting positive self-oriented and social well-being items—suggests that it is uniquely involved in evaluations that go ‘against the grain’. This dovetails with recent work describing a network-based model of self-concept structure, showing that greater vmPFC activity is negatively associated with a measure (outdegree centrality) implicated in favorable and coherent self-evaluations (Elder *et al.*, 2023).

Overall, these results are consistent with the hypothesis that the pgACC and vmPFC are not only engaged during self-reflection but also play a direct role in evaluation (Shenhav and Karmarkar, 2019; Barendse *et al.*, 2020) and the construction of individuals’ subjective sense of psychological health and well-being. Consistent with other research studies examining the pgACC and vmPFC within the same statistical models, their roles are complementary but distinctive (Shenhav and Karmarkar, 2019). Prior work on the neural correlates of self-referential processing that were not powered for such granular assessment on a trial-by-trial basis (e.g. due to small samples or collapsing across trials in univariate analyses) has tended to view these frontal midline components as relatively interchangeable, but this study reveals the nuance in how this network collectively supports intuitive access to a subjective sense of well-being at a given moment. Furthermore, these results highlight the importance of considering brain regions in concert rather than merely in isolation for identifying dissociability and adding depth to our understanding of functional brain networks.

### Limitations and future directions

Despite notable strengths, such as extending self-evaluation beyond traits to examine personal psychological health and assessing trial-level relationships between the brain and behavior in ways that enable the identification of unique functional relationships, these results should be considered in light of several limitations. First, the sample consisted solely of incoming college freshmen. Although these results are consistent with findings from a sample of 10–13-year-old adolescents (Barendse *et al.*, 2020), future studies should examine these relationships with a broader age range and more diverse, non-college samples. Second, the task design prohibited us from examining the unique relationships between psychological health constructs and valence as pre-registered. Supplementary analyses including valence only directly parallel the results reported in the main paper, but future studies that are specifically designed to test these distinctions would help illuminate potential differences.

Finally and critically, this study was conceptualized within a relatively White, Western and individualistic framework of psychological health that assumes universality (Christopher and Hickinbottom, 2008), but neglects systemic factors that have historically excluded specific groups, such as enslaved Africans and their descendants, and continue to limit and shape opportunities for well-being. In fact, some scholars have argued that psychological health as a construct is fundamentally anti-Black (Patterson, 1982). We recognize the role that psychological science has played in pathologizing, oppressing and incarcerating Black and other racially minoritized people (Auguste *et al.*, 2023; American Psychological Association, 2021). Therefore, we urge researchers to critically interrogate predominant models of psychological health and well-being and incorporate a broader range of perspectives that acknowledge these historical and cultural contexts (Christopher and Howe, 2014; Dudgeon, 2020; Johnson and Carter, 2020; Mosley *et al.*, 2020).

## Conclusions

A complete picture of whether someone is ‘doing well’ includes more than merely the absence of mental illness, but also positive functioning and social connectedness. The present study extended current methods for studying the neurobiological basis of psychological health and well-being by examining the neural correlates of self-evaluated psychological health and their dynamic relationship with trial-level evaluations of well-being. Overall, our results indicated strong consistency across constructs in the network of brain regions engaged by self-reflection and suggest that the pgACC and vmPFC play direct yet distinct roles in subjective evaluation.

## Supplementary Material

nsad065_Supp

## Positionality statement

In acknowledgment that our identities influence our approach to science (Roberts *et al.*, 2020), we wish to provide the reader with information about our backgrounds. At the time this paper was drafted, one author self-identified as an Iranian-American man of color, one author self-identified as a White, cis-gendered and heterosexual woman and one author self-identified as a White, cis-gendered and heterosexual woman who is a first-generation scholar and second-generation American. These identities, in combination with our experiences living in and receiving training in psychological science in the USA, have shaped our conceptualizations of psychological health and well-being, belonging and social capital in late adolescents’ transition to college.
