# Supplementary material for: If you’re happy and you know it: neural correlates of self-evaluated psychological health and well-being
Source: Soc Cogn Affect Neurosci. 2023 Nov 1;18(1):nsad065. doi: 10.1093/scan/nsad065 (PMC10684270; doi:10.1093/scan/nsad065)
Supplement: nsad065_Supp [file nsad065_supp.zip › scan-22-242-File002.docx]

**Supplementary material**

If you’re happy and you know it:

Neural correlates of self-evaluated psychological health and well-being

Danielle Cosme*

Arian Mobasser*

Jennifer H. Pfeifer

**Participant recruitment and inclusion criteria**

Participants indicated interest in participating in this paid research study while registering for summer orientation, and were subsequently contacted and screened for eligibility. Participants were eligible if they were incoming freshmen between 17-19 years old, right-handed, had not previously attended college at a different institution, and were planning to live on campus during their first year of college. Potential participants were not enrolled if they endorsed one or more of the following items: diagnosis of a psychiatric, learning, or neurologic disorder; presence of disordered eating or diagnosis of a condition that significantly impacted their diet; use of psychotropic medications; significant visual impairment or color blindness; concussion or other brain trauma; MRI contraindications (e.g. metal implants, biomedical devices, pregnancy).

**Self-evaluation task stimuli**

Well-being is conceptualized in a variety of ways in the literature, but there is consensus that well-being is comprised of positive states that contribute to overall psychological health independently of negative states, or ill-being. In other words, well-being and ill-being are related but distinct constructs and not merely opposite ends of a single continuum. Furthermore, social connectedness also exerts independent effects on psychological health. With these findings in mind, we adopted a data-driven approach to generate the stimuli used in the self-evaluation task (described below) and conducted a factor analyses using data from an independent sample of college students (N = 110, 63 females, as defined by sex assigned at birth). Participants rated 170 items taken from various mental health and well-being questionnaires, including: PERMA-Profiler [(Butler & Kern, 2016)](https://www.zotero.org/google-docs/?rGKxl7), Psychological Well-being [(Ryff & Keyes, 1995)](https://www.zotero.org/google-docs/?azLkSy), Positive Youth Development [(Geldhof et al., 2014)](https://www.zotero.org/google-docs/?4XvuqU), EPOCH Model of Adolescent Wellbeing [(Kern et al., 2016)](https://www.zotero.org/google-docs/?9SO7ZJ), Perceived Stress Scale [(Cohen et al., 1983)](https://www.zotero.org/google-docs/?WsI61l), and Center for Epidemiological Studies–Depression, 10 item [(Andresen et al., 1994)](https://www.zotero.org/google-docs/?rC07zr). For each item, participants rated how true each item was for them on a 4-point Likert-type scale (1 = Not at all true, 4 = Extremely true). We then conducted a series of factor analyses using the psych package [(Revelle, 2018)](https://www.zotero.org/google-docs/?A1O2gB) in R 3.5.1 [(R Core Team, 2018](https://www.zotero.org/google-docs/?SmUWAR); https://www.r-project.org/) to select items for the self-evaluation task. The goal of these analyses was to create three unique components related to psychological health to use in the self-evaluation MRI task. First, to explore the structure of the data, we ran a principal component analysis without rotation and extracted 6 components based on visual inspection of a scree plot of the eigenvalues. This analysis revealed components relating to well-being and ill-being, but the other components were less clearly labeled. In addition, for all but the well-being factor, the factor loadings were mediocre (highest loadings < 0.58). Consequently, we chose to allow items to correlate and ran a principal component analysis using a promax rotation. We first selected three components, which resulted in components related to well-being, ill-being, and physical health. Because we wanted to focus on psychological health and well-being, we then specified four components, which yielded components related to self-oriented well-being, social well-being, ill-being, and physical health (not of interest). We then used the factor loadings to select 20-40 items for each of the three components of interest and included reverse coded items in the social well-being component. We vetted the items for uniqueness, clarity, and readability, and selected 18 distinct items for each component to be used in the self-evaluation task. Item loadings and analytic code from this analysis and all other analyses reported here can be found online(https://github.com/dsnlab/happy_scripts). In this sample, the constructs were correlated as follows: self-oriented well-being and social well-being: r = .62 [.50, .73]; self-oriented well-being and ill-being: r = -.62 [-.75, .48]; social well-being and ill-being: r = -.29 [-.50, -.08].

**Stimuli used in the self-evaluation task**

**Self-oriented well-being.** Happy, satisfied with life, live a purposeful life, joyful, achieve goals, optimistic, live a meaningful life, hopeful, love life, interested in life, have direction, capable, positive, like myself, cheerful, proud, full of energy, engaged in tasks

**Social well-being.** Am cared for, comfortable with friends, have good friends, loved, trusted, have support systems, have warm relationships, have trusting relationships, feel supported, helped by friends, help others, well-liked, belong in social group, feel encouraged, alienated, isolated, neglected, lonely

**Ill-being.** Anxious, overwhelmed, nervous, unable to cope, feel rejected, depressed, stressed, sad, afraid of being hurt, feel worthless, worn out, tired, fearful, feel unimportant, unhappy, angry, feel invisible, feel useless

The percentage of trials in which each item was endorsed are listed in Table S1.

| Table S1  *Percent endorsements for items used in the self-evaluation task.* | | | |
| --- | --- | --- | --- |
| Construct | Item | *N* | % |
| self-oriented well-being* | capable | 5 / 5 | 100.0 |
| ill-being* | tired | 5 / 5 | 100.0 |
| social well-being* | have support systems | 5 / 5 | 100.0 |
| social well-being* | well-liked | 5 / 5 | 100.0 |
| self-oriented well-being | interested in life | 97 / 98 | 99.0 |
| social well-being | loved | 102 / 103 | 99.0 |
| social well-being | trusted | 101 / 104 | 97.1 |
| social well-being | am cared for | 98 / 102 | 96.1 |
| social well-being | comfortable with friends | 96 / 104 | 92.3 |
| self-oriented well-being | happy | 94 / 102 | 92.2 |
| self-oriented well-being | live a meaningful life | 95 / 103 | 92.2 |
| self-oriented well-being | achieve goals | 95 / 104 | 91.3 |
| social well-being | have warm relationships | 95 / 104 | 91.3 |
| self-oriented well-being | hopeful | 88 / 97 | 90.7 |
| self-oriented well-being | live a purposeful life | 92 / 102 | 90.2 |
| self-oriented well-being | engaged in tasks | 87 / 97 | 89.7 |
| social well-being | have good friends | 90 / 102 | 88.2 |
| social well-being | have trusting relationships | 87 / 99 | 87.9 |
| self-oriented well-being | joyful | 88 / 103 | 85.4 |
| self-oriented well-being | like myself | 84 / 103 | 81.6 |
| social well-being | feel encouraged | 82 / 101 | 81.2 |
| self-oriented well-being | positive | 79 / 98 | 80.6 |
| self-oriented well-being* | full of energy | 4 / 5 | 80.0 |
| self-oriented well-being* | optimistic | 4 / 5 | 80.0 |
| social well-being | helped by friends | 76 / 96 | 79.2 |
| self-oriented well-being | satisfied with life | 79 / 101 | 78.2 |
| self-oriented well-being | proud | 78 / 100 | 78.0 |
| ill-being | stressed | 67 / 100 | 67.0 |
| social well-being | belong in social group | 62 / 95 | 65.3 |
| self-oriented well-being* | cheerful | 3 / 5 | 60.0 |
| ill-being | anxious | 57 / 104 | 54.8 |
| ill-being | nervous | 53 / 103 | 51.5 |
| ill-being | overwhelmed | 49 / 103 | 47.6 |
| social well-being | lonely | 31 / 102 | 30.4 |
| ill-being | fearful | 26 / 98 | 26.5 |
| ill-being* | unhappy | 1 / 4 | 25.0 |
| social well-being | isolated | 24 / 102 | 23.5 |
| ill-being | feel unimportant | 18 / 103 | 17.5 |
| ill-being | feel rejected | 17 / 104 | 16.3 |
| ill-being | sad | 14 / 99 | 14.1 |
| ill-being | angry | 10 / 98 | 10.2 |
| ill-being | feel useless | 9 / 99 | 9.1 |
| ill-being | unable to cope | 8 / 104 | 7.7 |
| ill-being | feel worthless | 5 / 99 | 5.1 |
| ill-being* | depressed | 0 / 4 | 0.0 |
| ill-being* | feel invisible | 0 / 5 | 0.0 |
| social well-being* | alienated | 0 / 5 | 0.0 |
| social well-being* | neglected | 0 / 5 | 0.0 |
| *Note*. 12 items (4 per construct; flagged with *) were improperly randomized, resulting in far fewer participants seeing these items. Consequently, interpretation of the percent endorsements for these items should be done with caution. | | | |

**Neuroimaging data acquisition and preprocessing**

We acquired a high-resolution anatomical T1-weighted MP-RAGE scan (TR/TE = 2500.00/3.43ms, 256 × 256 matrix, 1mm thick, 176 sagittal slices, FOV = 208 × 208mm), functional images with a T2*- weighted echo-planar sequence (72 axial slices, TR/TE = 2000.00/25.00ms, 90-degree flip angle, 104 × 104 matrix, 2mm thick, FOV = 208 × 208mm), and opposite phase encoded echo-planar images to correct for magnetic field inhomogeneities (72 axial slices, TR/TE = 6390.00/47.80ms, 90-degree flip angle, 104 ×104 matrix, 2mm thick, FOV = 208 × 208mm).

Neuroimaging data were preprocessed using fMRIPrep 1.1.4 [(Esteban et al., 2019](https://www.zotero.org/google-docs/?kQ5eM1), RRID:SCR_016216), which is based on Nipype 1.1.1 [(Gorgolewski et al., 2011; Gorgolewski et al., 2019)](https://www.zotero.org/google-docs/?OY3yCt), RRID:SCR_002502). The T1-weighted (T1w) image was corrected for intensity non-uniformity (INU) using N4BiasFieldCorrection [(Tustison et al., 2010](https://www.zotero.org/google-docs/?OGUP4K), ANTs 2.2.0), and used as T1w-reference throughout the workflow. The T1w-reference was then skull-stripped using antsBrainExtraction.sh (ANTs 2.2.0), using OASIS as target template. Brain surfaces were reconstructed using recon-all (FreeSurfer 6.0.1, RRID:SCR_001847, [Dale, Fischl, & Sereno, 1999)](https://www.zotero.org/google-docs/?V3gNg1), and the brain mask estimated previously was refined with a custom variation of the method to reconcile ANTs-derived and FreeSurfer-derived segmentations of the cortical gray-matter of Mindboggle ([Klein et al., 2009](https://www.zotero.org/google-docs/?VXZnTV), RRID:SCR_002438[)](https://www.zotero.org/google-docs/?CR7wip). Spatial normalization to the ICBM 152 Nonlinear Asymmetrical template version 2009c [(Fonov et al., 2009)](https://www.zotero.org/google-docs/?WO0qSX), RRID:SCR_008796) was performed through nonlinear registration with antsRegistration (ANTs 2.2.0, RRID:SCR_004757, [Avants, Epstein, Grossman, & Gee, 2008)](https://www.zotero.org/google-docs/?VaPFNp), using brain-extracted versions of both T1w volume and template. Brain tissue segmentation of cerebrospinal fluid (CSF), white-matter (WM) and gray-matter (GM) was performed on the brain-extracted T1w using fast (FSL 5.0.9, RRID:SCR_002823, [Zhang, Brady, & Smith, 2001)](https://www.zotero.org/google-docs/?mUGv4q).

For each of the two functional runs per subject (across all tasks and sessions), the following preprocessing was performed. First, a reference volume and its skull-stripped version were generated using a custom methodology of fMRIPrep. A deformation field to correct for susceptibility distortions was estimated based on two echo-planar imaging (EPI) references with opposing phase-encoding directions, using 3dQwarp (AFNI). Based on the estimated susceptibility distortion, an unwarped BOLD reference was calculated for a more accurate co-registration with the anatomical reference. Head-motion parameters with respect to the BOLD reference (transformation matrices and six corresponding rotation and translation parameters) are estimated before any spatiotemporal filtering using mcflirt (FSL 5.0.9, [Jenkinson, Bannister, Brady, & Smith, 2002)](https://www.zotero.org/google-docs/?jJQCDB). The BOLD time-series were resampled onto their original, native space by applying a single, composite transform to correct for head-motion and susceptibility distortions. These resampled BOLD time-series will be referred to as preprocessed BOLD in original space, or just preprocessed BOLD. The BOLD reference was then co-registered to the T1w reference using bbregister (FreeSurfer) which implements boundary-based registration [(Greve & Fischl, 2009)](https://www.zotero.org/google-docs/?Plg6sA). Co-registration was configured with nine degrees of freedom to account for distortions remaining in the BOLD reference. The BOLD time-series were resampled to surfaces in fsnative space. The BOLD time-series were resampled to MNI152NLin2009cAsym standard space, generating a preprocessed BOLD run in MNI152NLin2009cAsym space.

Several confounding time-series were calculated based on the preprocessed BOLD: framewise displacement (FD), DVARS and three region-wise global signals. FD and DVARS are calculated for each functional run, both using their implementations in Nipype (following the definitions by [Power et al., 2014)](https://www.zotero.org/google-docs/?EbNBuc). The three global signals are extracted within the CSF, the WM, and the whole-brain masks. Additionally, a set of physiological regressors were extracted to allow for component-based noise correction (CompCor; [Behzadi, Restom, Liau, & Liu, 2007)](https://www.zotero.org/google-docs/?H5KSR3). Principal components are estimated after high-pass filtering the preprocessed BOLD time-series (using a discrete cosine filter with 128s cut-off) for the two CompCor variants: temporal (tCompCor) and anatomical (aCompCor). Six tCompCor components are then calculated from the top 5% variable voxels within a mask covering the subcortical regions. This subcortical mask is obtained by heavily eroding the brain mask, which ensures it does not include cortical GM regions. For aCompCor, six components are calculated within the intersection of the aforementioned mask and the union of CSF and WM masks calculated in T1w space, after their projection to the native space of each functional run (using the inverse BOLD-to-T1w transformation). The head-motion estimates calculated in the correction step were also placed within the corresponding confounds file. All resamplings can be performed with a single interpolation step by composing all the pertinent transformations (i.e., head-motion transform matrices, susceptibility distortion correction when available, and co-registrations to anatomical and template spaces). Gridded (volumetric) resamplings were performed using antsApplyTransforms (ANTs), configured with Lanczos interpolation to minimize the smoothing effects of other kernels [(Lanczos, 1964)](https://www.zotero.org/google-docs/?pViiaE). Non-gridded (surface) resamplings were performed using mri_vol2surf (FreeSurfer). Many internal operations of fMRIPrep use Nilearn 0.4.2 [(Abraham et al., 2014](https://www.zotero.org/google-docs/?O2LjY5), RRID:SCR_001362), mostly within the functional processing workflow. For more details of the pipeline, see [the section corresponding to workflows in fMRIPrep’s documentation](https://fmriprep.readthedocs.io/en/latest/workflows.html).

**ROI definition**

We defined three a priori bilateral ROIs for pgACC, vmPFC, and VS (see Figure 3) using the HCP MMP 1.0 cortical parcellation atlas (Glasser et al., 2016) and the FreeSurfer segmentation atlas (Fischl et al., 2002). These ROIs were mapped to participants’ T1-weighted anatomical scans using FreeSurfer 6 (Fischl, 2012). Cortical ROIs were specified by identifying parcels that overlapped with the medial prefrontal cluster from the group-level self > change contrast and were divided into vmPFC (including L_p32_ROI, L_10r_ROI, and L_10v_ROI parcels) and pgACC ROIs (including L_a24_ROI, L_25_ROI, and L_s32_ROI parcels) based on anatomical location. The VS ROI was defined by combining the nucleus accumbens and putamen segmentations. All ROIs were concatenated and binarized using the fslmaths function in FSL 5.0.10 (Jenkinson, Beckmann, Behrens, Woolrich, & Smith, 2012). This process yielded individually defined pgACC, vmPFC, and VS ROIs for each participant in native space.

**Preregistered analyses**

***Trial-level analyses without reaction time***

We included reaction time in the model reported in the main manuscript to control for its confounding effects. However, the original preregistered model did not include reaction time. For transparency, we report the results without reaction time in Table S2. These results are directionally consistent with the results including reaction time; all terms except the interaction between vmPFC and construct (ill-being) tended to be weaker and were not statistically significant.

| Table S2  *Parameter estimates and statistics for the preregistered model not including reaction time* | | | |
| --- | --- | --- | --- |
| Parameter | *b* | *z* | *p* |
| Intercept (well-being) | 2.01 [1.81, 2.21] | 19.89 | < .001 |
| Construct (ill-being) | -3.06 [-3.29, -2.83] | 26.15 | < .001 |
| Construct (social) | -0.09 [-0.34, 0.15] | 0.74 | .460 |
| pgACC | 0.25 [-0.06, 0.55] | 1.60 | .110 |
| vmPFC | -0.23 [-0.53, 0.08] | 1.47 | .142 |
| VS | 0.13 [-0.05, 0.30] | 1.40 | .161 |
| Construct (ill-being) x pgACC | -0.19 [-0.58, 0.19] | 0.99 | .321 |
| Construct (social) x pgACC | 0.02 [-0.40, 0.43] | 0.08 | .934 |
| Construct (ill-being) x vmPFC | 0.59 [0.20, 0.98] | 2.98 | .003 |
| Construct (social) x vmPFC | -0.08 [-0.50, 0.35] | 0.37 | .715 |
| Construct (ill-being) x VS | -0.19 [-0.43, 0.04] | 1.61 | .106 |
| Construct (social) x VS | -0.08 [-0.32, 0.17] | 0.62 | .533 |
| *Note.* Parameter estimates (*b*) are log-odds. Neural ROI parameter estimates were standardized within participant and ROI to preserve individual differences and are therefore not *Z*-scores. | | | |

***Trial-level similarity analysis***

Here we report results from the preregistered similarity analysis conducted on a trial-by-trial basis. Please note that we report these results for the sake of completeness and fidelity to the preregistration; these estimates are likely to be unstable because the task design was not optimized for similarity analysis [(Zeithamova et al., 2017)](https://www.zotero.org/google-docs/?fJTbsB). For each participant, we extracted the patterns of activity for each *a priori* ROI (pgACC, vmPFC, VS) on a trial-by-trial basis. To do this, we created a “beta-series” by running another first-level GLM that modeled each trial as a separate regressor [(Rissman et al., 2004)](https://www.zotero.org/google-docs/?0yvz04). We included the same motion regressors as described in the main manuscript. From the 72 beta maps created, we selected the 36 maps for the trials in the self condition (18 in each run) and extracted the parameter estimates for each voxel in each ROI. Because trials within runs are highly correlated, we calculated trial correlations across runs (i.e., trials in run 1 were only correlated with trials in run 2). In each run there are 18 items in the self condition, so for each participant, we generated 324 correlations for each ROI. Correlations were then converted to Z values using a Fisher Z-transformation and averaged across participants for each construct pair and ROI. As shown in Figure S1, none of these variables were correlated on a trial-by-trial basis with this task design.


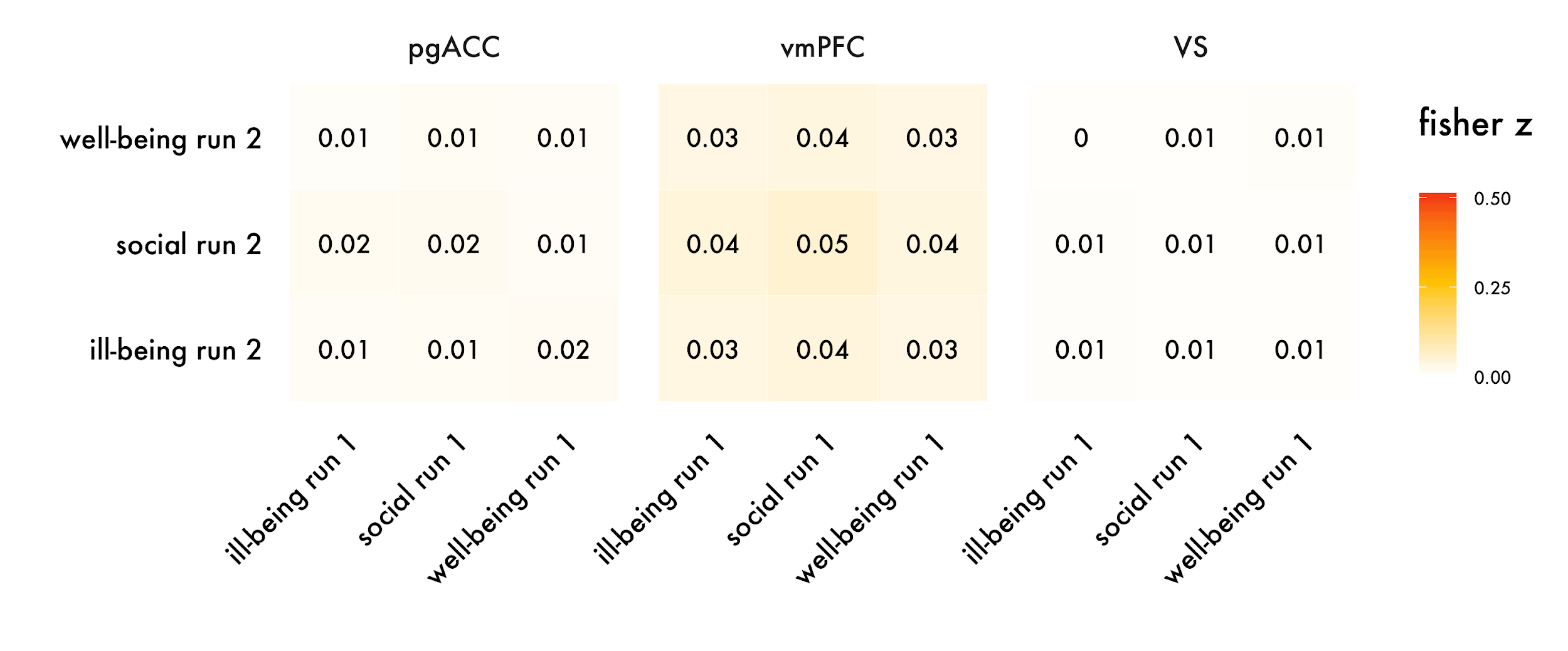


*Figure S1.* Mean Fisher Z-transformed correlations between task runs as a function of psychological health construct, calculated within the self condition only on a trial-by-trial basis.

***Individual difference analyses***

**Psychological health scores.** To extend the trial-level analyses which assessed the degree to which BOLD signal within ROIs involved in self-evaluation was associated with responses to each item during the task, we sought to estimate the extent to which mean BOLD signal (across trials) within these same ROIs was related to individual differences in psychological health. In other words, do neural predictors contribute additional information about individual differences in psychological health or do they only relate to specific evaluations made by an individual in the moment? To answer this question, we operationalized psychological health scores as the percentage of items endorsed within each construct (self-oriented well-being, social well-being, ill-being). The social well-being construct contained both positive and negatively valenced items, whereas the well-being construct only included positively valenced items and the ill-being construct only included negatively valenced items. Therefore, within the social well-being construct, psychological health scores were calculated as the sum of “yes” responses on positively valenced items and “no” responses on negatively valenced items. Consequently, more “yes” responses in the social and self-oriented well-being constructs indicates greater psychological health, whereas more “yes” responses in the ill-being construct indicates lower psychological health. To control for differences in the number of missed responses across participants, we divided scores by the total number of responses in each construct and multiplied by 100 to create percentages.

**Behavioral analysis.** We investigated the effect of task Instruction (self or change) and Construct (self-oriented well-being, social well-being, ill-being) on psychological health scores using multilevel modeling in R using the lmer function in the lme4 package (Bates et al., 2015). We regressed psychological health scores on the fixed effects of instruction and construct and modeled participant intercepts as random effects.

**Neural analysis.** To determine the degree to which mean BOLD signal in ROIs implicated in self-evaluation and affective processing is associated with psychological health scores, we extracted mean parameter estimates within ROIs from the self > rest contrasts for each construct. Because this analysis is a between subjects comparison, we used models and ROIs in MNI space rather than native space. Our preregistered plan was to fit a series of nested multilevel models regressing psychological health scores on the fixed effects of construct, ROI, and their interaction, adding ROIs and interaction terms in the following stepwise fashion (as in the trial-level analyses). We planned to then use chi-square difference tests to assess whether each successive model improved model fit to the observed data. The order of nested models was determined a priori based on our hypothesis that pgACC would be associated with psychological health scores, whereas vmPFC and VS would not be. However, none of these models converged. In order to still test this question, we used linear regression regressing psychological health scores on the ROIs for each well-being construct separately.

**Behavioral results.** We estimated the effect of Instruction (self or change) and Construct (self-oriented well-being, social well-being, ill-being) on the percentage of trials endorsed using multilevel modeling. Overall, this model fit the observed data well (R^2^ = 0.59). In line with previous research, we found a self-enhancement effect (Figure S2) on self-evaluations such that participants endorsed more items in the positively valenced self-oriented well-being (*M* = 86.94%, *SD* = 16.21) and social well-being (*M* = 87.18%, *SD* = 15.36) constructs than in the negatively valenced ill-being construct (*M* = 29.09%, *SD* = 21.00). We also observed that malleability ratings differed as a function of construct. Specifically, participants rated social well-being items as less malleable than both ill-being items (*M_diff_* = -14.98%) and self-oriented well-being items (*M_diff_* = -13.54%). Malleability ratings for ill-being items did not differ from self-oriented well-being items (*M_diff_* = 1.44%). All parameter estimates from this model and relevant statistics can be found in Table S3.

**
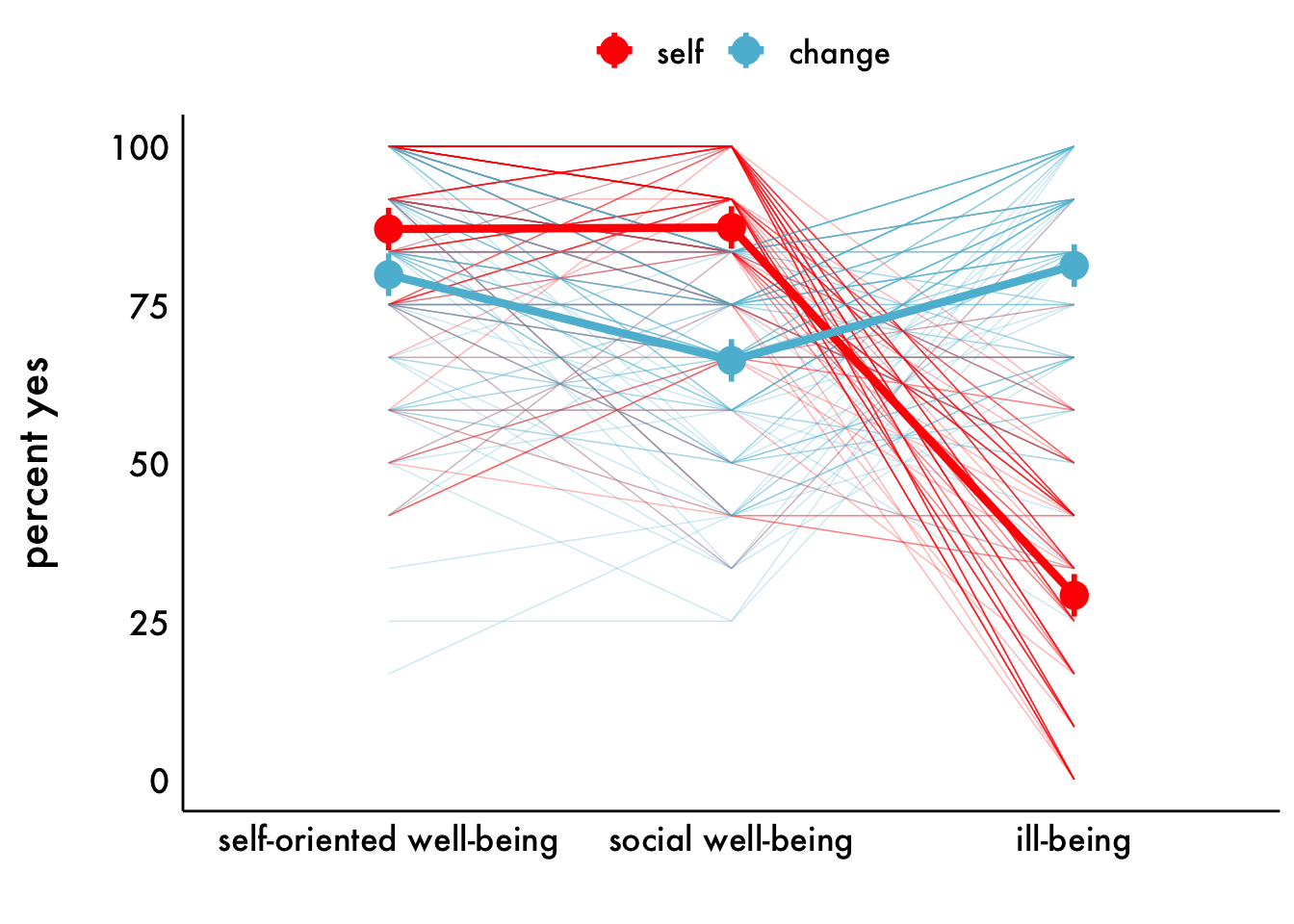
**

*Figure S2*. Percentage of responses in which participants responded yes as a function of construct and instruction. Thin lines represent individual values, whereas thick lines represent the group mean. Error bars are 95% confidence intervals.

| Table S3  *Parameter estimates and statistics for behavioral psychological health and malleability scores* | | | | |
| --- | --- | --- | --- | --- |
| Parameter | *b* [95% CI] | *df* | *t* | *p* |
| Intercept | 79.73 [76.37, 83.09] | 612.7 | 46.59 | < .001 |
| Construct (ill-being) | 1.44 [-3.21, 6.10] | 515.0 | 0.61 | .543 |
| Construct (social well-being) | -13.54 [-18.20, -8.89] | 515.0 | 5.72 | < .001 |
| Instructions (self) | 7.21 [2.56, 11.87] | 515.0 | 3.04 | .002 |
| Construct (ill-being) x Instructions (self) | -59.29 [-65.88, -52.71] | 515.0 | 17.70 | < .001 |
| Construct (social well-being) x Instructions (self) | 13.78 [7.20, 20.36] | 515.0 | 4.11 | < .001 |
| *Note.* Parameter estimates (*b*) are percentages. Higher percentages indicate greater self-oriented well-being, social well-being, and ill-being. Degrees of freedom (*df*) were calculated using the Satterthwaite approximation. | | | | |

**Neural results.** Contrary to our hypotheses, none of the ROIs had statistically significant relationships with the psychological health and well-being scores (Table S4; Figure S3).

| Table S4  *Parameter estimates and statistics for the neural psychological health and malleability scores models* | | | |
| --- | --- | --- | --- |
| Parameter | *b* [95% CI] | *t* | *p* |
| Self-oriented well-being | | | |
| Intercept | 86.24 [82.27, 90.20] | 43.16 | < .001 |
| pgACC | 4.93 [-1.67, 11.52] | 1.48 | .141 |
| vmPFC | -3.11 [-10.11, 3.90] | 0.88 | .381 |
| VS | 1.01 [-3.37, 5.39] | 0.46 | .647 |
|  |  |  |  |
| Social well-being | | | |
| Intercept | 86.47 [82.46, 90.49] | 42.74 | < .001 |
| pgACC | -1.27 [-7.43, 4.89] | 0.41 | .683 |
| vmPFC | 1.14 [-4.01, 6.29] | 0.44 | .662 |
| VS | 1.73 [-1.69, 5.15] | 1.01 | .317 |
|  |  |  |  |
| Ill-being | | | |
| Intercept | 30.79 [25.88, 35.70] | 12.44 | < .001 |
| pgACC | -3.15 [-12.74, 6.45] | 0.65 | .517 |
| vmPFC | 3.04 [-6.35, 12.43] | 0.64 | .522 |
| VS | -2.98 [-7.73, 1.76] | 1.25 | .215 |
| *Note.* Parameter estimates (*b*) are percentages. Higher percentages indicate greater self-oriented well-being, social well-being, and ill-being. | | | |


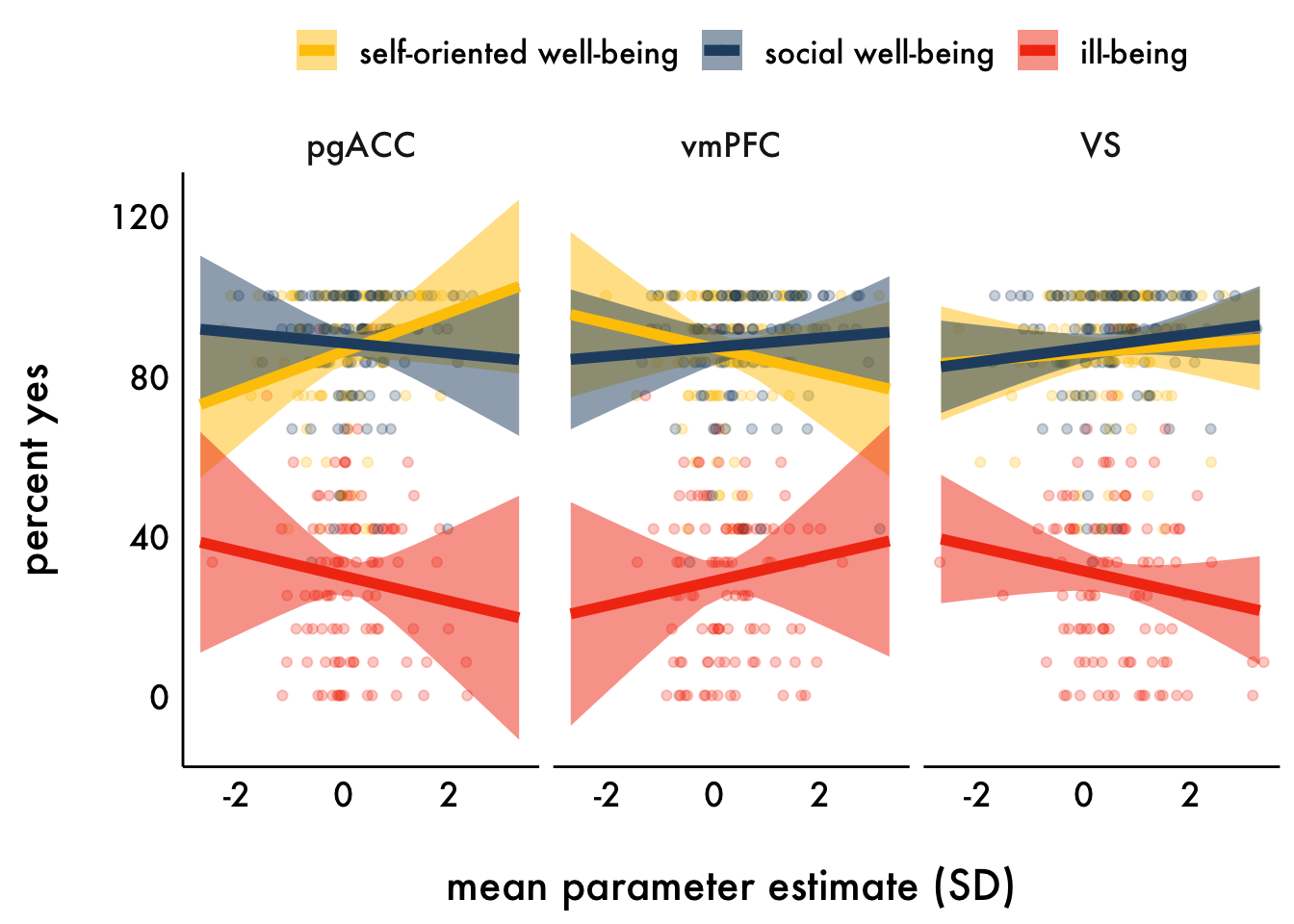


*Figure S3*. Predicted psychological health scores (percent endorsements) as a function of construct and ROI. Points represent individual scores, and lines represent group means. Error bands are 95% confidence intervals.

**Sensitivity and post-hoc analyses**

***Trial-level analyses using events with a 2s duration***

We conducted a sensitivity analysis in which we used a fixed duration of 2s rather than reaction time in first-level fMRI models. We chose 2s since the mean reaction time across all trials was 1.62s and 80% of trials had reaction times less than 2 seconds. We extracted parameter estimates from these models and conducted the trial-level analyses reported in the main manuscript. In general, these results reported in Tables S5-6 mirror the primary analysis.”

| Table S5  *Parameter estimates and statistics for the model including log-transformed reaction time using events with 2s duration* | | | |
| --- | --- | --- | --- |
| Parameter | *b* | *z* | *p* |
| Intercept (self-oriented well-being) | 1.92 [1.70, 2.14] | 16.98 | < .001 |
| RT | -3.20 [-3.80, -2.61] | 10.53 | < .001 |
| Construct (social well-being) | 0.08 [-0.21, 0.36] | 0.52 | .605 |
| Construct (ill-being) | -2.96 [-3.21, -2.71] | 23.48 | < .001 |
| pgACC | 0.36 [0.04, 0.69] | 2.20 | .028 |
| vmPFC | -0.45 [-0.78, -0.13] | 2.77 | .006 |
| VS | 0.14 [-0.06, 0.34] | 1.37 | .170 |
| RT x Construct (social well-being) | 0.05 [-0.74, 0.84] | 0.13 | .895 |
| RT x Construct (ill-being) | 3.21 [2.50, 3.91] | 8.91 | < .001 |
| Construct (social well-being) x pgACC | 0.01 [-0.42, 0.43] | 0.04 | .967 |
| Construct (ill-being) x pgACC | -0.20 [-0.59, 0.19] | 1.00 | .316 |
| Construct (social well-being) x vmPFC | -0.10 [-0.53, 0.32] | 0.47 | .637 |
| Construct (ill-being) x vmPFC | 0.72 [0.33, 1.11] | 3.60 | < .001 |
| Construct (social well-being) x VS | -0.06 [-0.33, 0.21] | 0.42 | .674 |
| Construct (ill-being) x VS | -0.29 [-0.54, -0.05] | 2.32 | .020 |
| *Note.* Parameter estimates (*b*) are log-odds. RTs were grand-mean centered and units are in seconds. Neural ROI parameter estimates were standardized within participant and ROI to preserve individual differences and are therefore not *Z*-scores. | | | |

| Table S6  *Parameter estimates and statistics for the preregistered model not including reaction time using events with 2s duration* | | | |
| --- | --- | --- | --- |
| Parameter | *b* | *z* | *p* |
| Intercept (self-oriented well-being) | 2.06 [1.86, 2.26] | 19.91 | < .001 |
| Construct (social well-being) | -0.07 [-0.33, 0.19] | 0.51 | .609 |
| Construct (ill-being) | -3.10 [-3.33, -2.86] | 26.05 | < .001 |
| pgACC | 0.36 [0.06, 0.67] | 2.37 | .018 |
| vmPFC | -0.40 [-0.70, -0.10] | 2.61 | .009 |
| VS | 0.11 [-0.08, 0.29] | 1.14 | .255 |
| Construct (social well-being) x pgACC | 0.00 [-0.40, 0.40] | 0.01 | .995 |
| Construct (ill-being) x pgACC | -0.21 [-0.58, 0.17] | 1.09 | .276 |
| Construct (social well-being) x vmPFC | -0.12 [-0.52, 0.28] | 0.58 | .564 |
| Construct (ill-being) x vmPFC | 0.66 [0.29, 1.04] | 3.48 | < .001 |
| Construct (social well-being) x VS | -0.05 [-0.30, 0.20] | 0.39 | .698 |
| Construct (ill-being) x VS | -0.26 [-0.50, -0.03] | 2.17 | .030 |
| *Note.* Parameter estimates (*b*) are log-odds. Neural ROI parameter estimates were standardized within participant and ROI to preserve individual differences and are therefore not *Z*-scores. | | | |

***Trial-level analyses with valence***

Although we were unable to model construct and valence in the same model as preregistered, we conducted a follow-up analysis modeling valence only. Overall, this model fit worse than the construct model reported in the main manuscript (AIC_valence_ = 3223.89; AIC_construct_ = 3082.28), suggesting that the construct model provides additional information not captured in valence alone. In general, the results (Table S7; Figure S4) from this model largely mirror those in the construct model. The same pattern of results are present and are generally stronger in magnitude for the valence models. For example, whereas the negative interactions between ill-being and pgACC and VS were not statistically significant in the construct model, they are stronger in magnitude and are statistically significant in the valence model. Overall, regardless of whether modeling construct or valence, we observe the same differential relationships with self-evaluations for the different ROIs.

| Table S7  *Parameter estimates and statistics for the model including valence* | | | |
| --- | --- | --- | --- |
| Parameter | *b* | *z* | *p* |
| Intercept (well-being) | 2.06 [1.88, 2.25] | 22.11 | < .001 |
| RT | -3.31 [-3.76, -2.87] | 14.52 | < .001 |
| Valence (negative) | -2.79 [-2.99, -2.59] | 26.96 | < .001 |
| pgACC | 0.56 [0.28, 0.84] | 3.93 | < .001 |
| vmPFC | -0.58 [-0.86, -0.29] | 3.99 | < .001 |
| VS | 0.11 [-0.06, 0.28] | 1.24 | .215 |
| RT x Valence (negative) | 2.89 [2.34, 3.44] | 10.26 | < .001 |
| Valence (negative) x pgACC | -0.48 [-0.83, -0.13] | 2.72 | .006 |
| Valence (negative) x vmPFC | 0.93 [0.57, 1.28] | 5.13 | < .001 |
| Valence (negative) x VS | -0.24 [-0.45, -0.02] | 2.15 | .032 |
| *Note.* Parameter estimates (*b*) are log-odds. RTs were grand-mean centered and units are in seconds. Neural ROI parameter estimates were standardized within participant and ROI to preserve individual differences and are therefore not *Z*-scores. | | | |


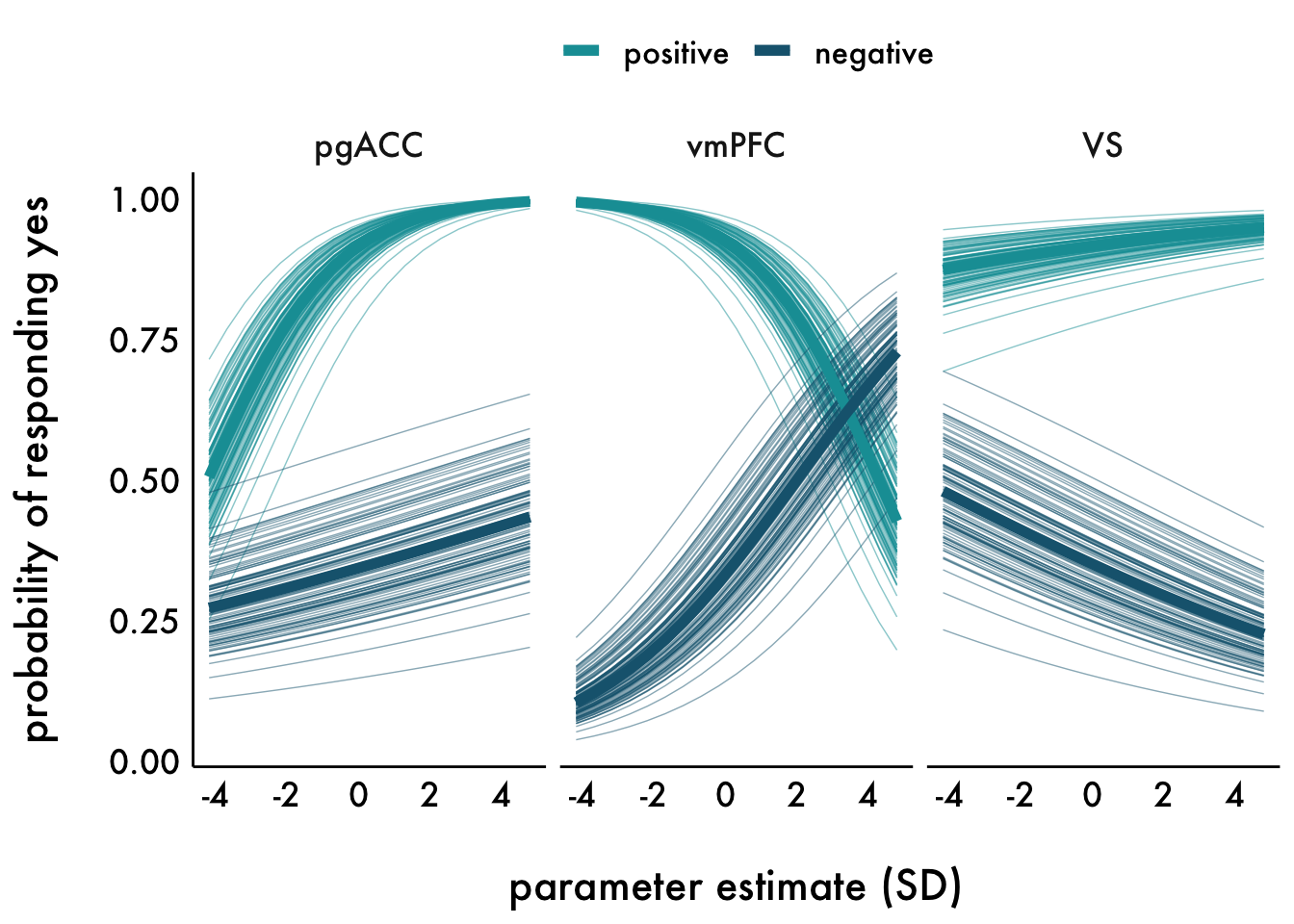


*Figure S4*. Predicted probabilities from the ROI interaction model as a function of valence. Thinner lines indicate the predicted probabilities for each participant as a function of valence and ROI. pgACC = perigenual anterior cingulate cortex, vmPFC = ventromedial prefrontal cortex, VS = ventral striatum.

***Trial-level analysis including item intercepts as a random effect***

Because items varied in how frequently they were endorsed (see Table S1), we conducted a sensitivity analysis including item intercepts as a random effect to account for item-level differences in endorsements. The results are reported in Table S8 and although the parameter estimates and statistical significance shifts slightly, the overall pattern of results is consistent with original analyses reported in the main manuscript.

| Table S8  *Parameter estimates and statistics for the model including item intercepts as a random effect* | | | |
| --- | --- | --- | --- |
| Parameter | *b* | *z* | *p* |
| Intercept (self-oriented well-being) | 1.97 [1.36, 2.57] | 6.38 | < .001 |
| RT | -3.51 [-4.14, -2.89] | 10.99 | < .001 |
| Construct (social well-being) | 0.41 [-0.44, 1.26] | 0.95 | .341 |
| Construct (ill-being) | -3.24 [-4.07, -2.42] | 7.73 | < .001 |
| pgACC | 0.40 [0.03, 0.77] | 2.09 | .037 |
| vmPFC | -0.37 [-0.74, 0.00] | 1.93 | .053 |
| VS | 0.13 [-0.09, 0.36] | 1.15 | .250 |
| RT x Construct (social well-being) | 0.35 [-0.49, 1.18] | 0.82 | .415 |
| RT x Construct (ill-being) | 4.05 [3.29, 4.82] | 10.37 | < .001 |
| Construct (social well-being) x pgACC | 0.13 [-0.39, 0.65] | 0.50 | .615 |
| Construct (ill-being) x pgACC | -0.42 [-0.87, 0.04] | 1.79 | .073 |
| Construct (social well-being) x vmPFC | -0.23 [-0.76, 0.31] | 0.83 | .409 |
| Construct (ill-being) x vmPFC | 0.67 [0.21, 1.13] | 2.83 | .005 |
| Construct (social well-being) x VS | -0.07 [-0.39, 0.24] | 0.45 | .651 |
| Construct (ill-being) x VS | -0.13 [-0.41, 0.16] | 0.87 | .385 |
| *Note*. Parameter estimates (b) are log-odds. RTs were grand-mean centered and units are in seconds. Neural ROI parameter estimates were standardized within participant and ROI to preserve individual differences and are therefore not Z-scores. | | | |

***Trial-level analyses including Instruction***

We conducted sensitivity analyses to examine the specificity of the findings reported in the main manuscript to the process of self-evaluation. We did this by including Instruction and its interaction with each ROI to directly compare the magnitude of the relationships between ROI activity and task responses when participants made judgements about themselves (self condition) versus malleability (change condition). To facilitate interpretation and more directly test specificity between the self and change conditions, we fit models for each well-being construct separately. These results are reported in Table S9 and visualized in Figure S5.

For social well-being, there is specificity in pgACC and vmPFC, but not VS. For self-oriented well-being, change endorsements did not vary substantially as a function of brain activity in any of the ROIs; there were directional differences in slopes for self > change for pgACC and vmPFC, but they were not statistically significant. For ill-being, the slopes differed between self and change only in pgACC. Overall this suggests clear specificity across well-being constructs for pgACC, specificity for positively valenced well-being constructs in vmPFC, and no specificity in VS.

| Table S9  *Parameter estimates and statistics for the models including Instruction* | | | |
| --- | --- | --- | --- |
| Parameter | *b* | *z* | *p* |
| Self-oriented well-being model | | | |
| Intercept | 2.00 [1.75, 2.26] | 15.33 | < .001 |
| RT | -3.35 [-3.97, -2.73] | 10.58 | < .001 |
| Instructions (change) | 0.02 [-0.27, 0.32] | 0.16 | .875 |
| pgACC | 0.47 [0.11, 0.82] | 2.56 | .011 |
| vmPFC | -0.45 [-0.83, -0.08] | 2.41 | .016 |
| VS | 0.09 [-0.12, 0.31] | 0.85 | .398 |
| RT x Instructions (change) | 1.30 [0.53, 2.06] | 3.32 | < .001 |
| Instructions (change) x pgACC | -0.45 [-0.94, 0.04] | 1.81 | .071 |
| Instructions (change) x vmPFC | 0.46 [-0.06, 0.98] | 1.74 | .083 |
| Instructions (change) x VS | -0.13 [-0.43, 0.18] | 0.83 | .406 |
|  |  |  |  |
| Social well-being model | | | |
| Intercept | 1.95 [1.73, 2.17] | 17.21 | < .001 |
| RT | -2.97 [-3.51, -2.42] | 10.71 | < .001 |
| Instructions (change) | -1.24 [-1.49, -0.99] | 9.76 | < .001 |
| pgACC | 0.42 [0.09, 0.75] | 2.48 | .013 |
| vmPFC | -0.51 [-0.86, -0.16] | 2.88 | .004 |
| VS | 0.10 [-0.10, 0.30] | 0.95 | .341 |
| RT x Instructions (change) | 2.80 [2.16, 3.44] | 8.59 | < .001 |
| Instructions (change) x pgACC | -0.78 [-1.21, -0.35] | 3.55 | < .001 |
| Instructions (change) x vmPFC | 0.85 [0.40, 1.30] | 3.68 | < .001 |
| Instructions (change) x VS | -0.01 [-0.27, 0.24] | 0.10 | .918 |
|  |  |  |  |
| Ill-being model | | | |
| Intercept | -1.10 [-1.30, -0.91] | 11.25 | < .001 |
| RT | -0.12 [-0.54, 0.31] | 0.54 | .588 |
| Instructions (change) | 2.95 [2.69, 3.21] | 22.44 | < .001 |
| pgACC | 0.08 [-0.16, 0.32] | 0.68 | .498 |
| vmPFC | 0.34 [0.10, 0.59] | 2.72 | .007 |
| VS | -0.10 [-0.25, 0.06] | 1.24 | .214 |
| RT x Instructions (change) | -1.81 [-2.46, -1.15] | 5.4 | < .001 |
| Instructions (change) x pgACC | -0.41 [-0.82, -0.01] | 2.01 | .044 |
| Instructions (change) x vmPFC | -0.12 [-0.56, 0.31] | 0.57 | .572 |
| Instructions (change) x VS | 0.04 [-0.22, 0.29] | 0.30 | .761 |
| Note. Parameter estimates (b) are log-odds. RTs were grand-mean centered and units are in seconds. Neural ROI parameter estimates were standardized within participant and ROI to preserve individual differences and are therefore not Z-scores. | | | |


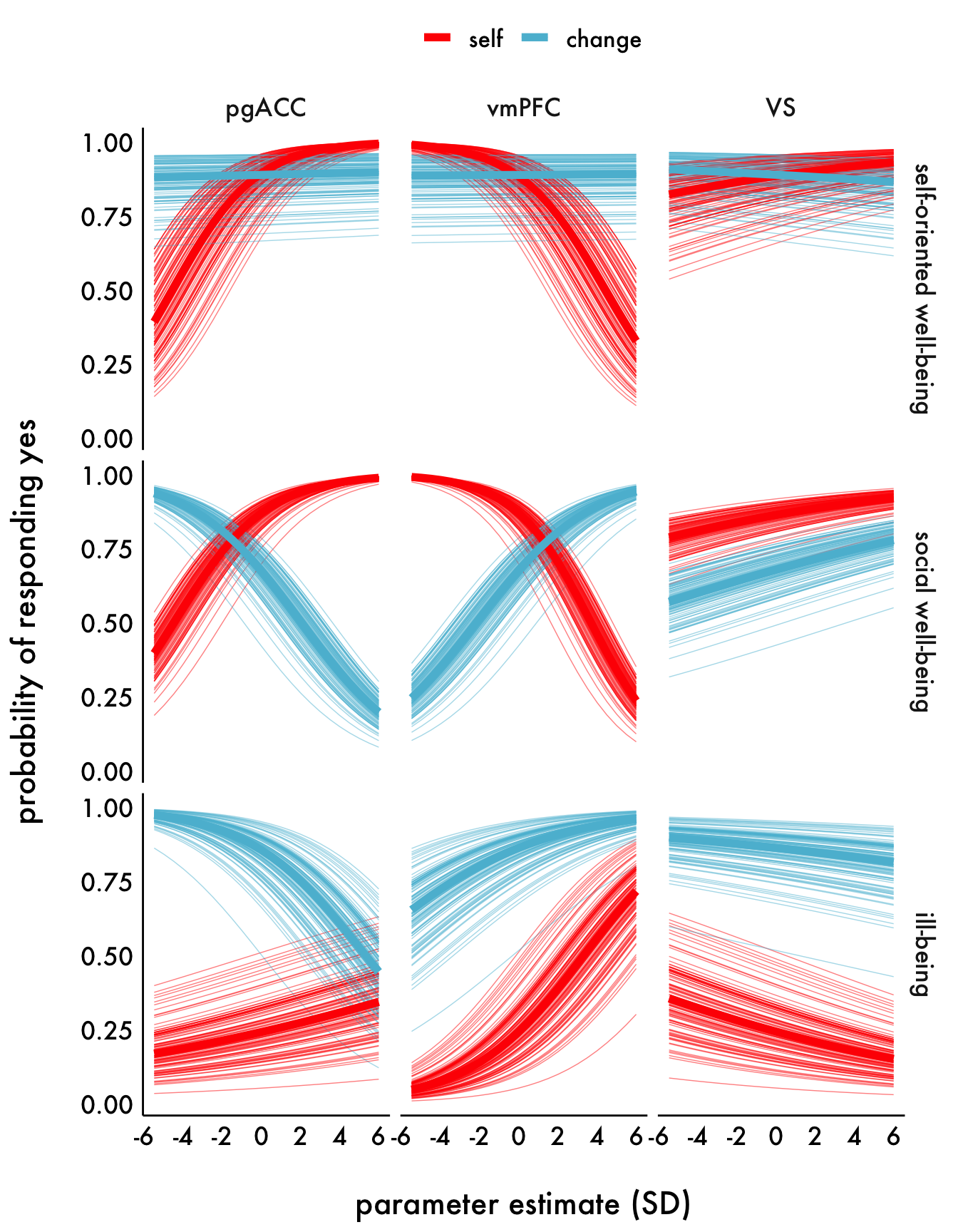


*Figure S5*. Predicted probabilities from the models reported in Table SX as a function of instruction, construct, and ROI. Thinner lines indicate the predicted probabilities for each participant as a function of instruction, construct, and ROI. pgACC = perigenual anterior cingulate cortex, vmPFC = ventromedial prefrontal cortex, VS = ventral striatum.
